# The Inauguration of State Editions of the “Journal”

**Published:** 1898-04

**Authors:** 


### EDITORIAL.

### THE INAUGURATION OF STATE EDITIONS OF THE "JOURNAL."

WITH this number we inaugurate a series of State Editions of the JOURNAL. We trust that this plan will meet the hearty co-operation of the profession throughout the country, and that the States desiring, in this way, to promote their interests professionally, scientifically, and socially will loan their best efforts in sustaining this movement.

Association officers and members will be given great latitude in their efforts to thus strengthen their organizations, and sanitary work and movements will be aided and promoted in proportion to the efforts put forth by those who should lead in this work. Our editorial columns will be open for all State subjects, and every phase of State work will be voiced in our columns. Legislation—State, municipal, and local—will be touched upon, and the opportunity thus afforded will be unique and, if well used, of the greatest advantage to all.

Extra editions will be issued each month, and our special State lists of veterinarians, physicians, boards of health, agricultural and breeders' association officers, and many others will each be favored with a copy, and we trust that much good will flow forth to all.

We issue this month the Pennsylvania Special, and May will be for the Empire State, and the other States as the material is contributed.

